# Network Pharmacology Analysis of Huangqi Jianzhong Tang Targets in Gastric Cancer

**DOI:** 10.3389/fphar.2022.882147

**Published:** 2022-04-08

**Authors:** Long Li, Yizhuo Lu, Yanling Liu, Dan Wang, Linshan Duan, Shuyu Cheng, Guoyan Liu

**Affiliations:** ^1^ School of Medicine, Xiamen University, Xiamen, China; ^2^ Department of General Surgery, Zhongshan Hospital of Xiamen University, School of Medicine, Xiamen University, Xiamen, China; ^3^ Institute of Gastrointestinal Oncology, School of Medicine, Xiamen University, Xiamen, China; ^4^ School of Pharmaceutical Sciences, Xiamen University, Xiamen, China; ^5^ Department of Gastrointestinal Surgery, Zhongshan Hospital of Xiamen University, School of Medicine, Xiamen University, Xiamen, China

**Keywords:** gastric cancer, Huangqi Jianzhong Tang, network pharmacology, molecular docking, molecular mechanism

## Abstract

**Background:** The Chinese medicine, Huangqi Jianzhong Tang (HJT), is widely used to treat gastric cancer (GC). In this study, network pharmacological methods were used to analyze the potential therapeutic targets and pharmacological mechanisms of HJT in GC.

**Methods:** Bioactive components and targets of HJT and GC-related targets were identified using public databases. The protein-protein interaction network of potential targets of HJT in GC was constructed using the Cytoscape plug-in (v3.8.0), CytoHubba. Gene Ontology (GO) and Kyoto Encyclopedia of Genes and Genomes (KEGG) pathway enrichment analyses were performed, in addition to molecular docking and animal experiments to verify the results of network pharmacology analysis.

**Results:** A total of 538 GC-related targets were identified. The bioactive components of HJT were selected for drug-likeness evaluation and binomial statistical model screening, which revealed 63 bioactive components and 72 targets. Based on GO enrichment analysis, all targets in the protein-protein interaction network were mainly involved in the response to oxidative stress and neuronal death. Further, KEGG enrichment analysis suggested that the treatment of GC with HJT mainly involved the Wnt signaling pathway, PI3K-Akt signaling pathway, TGF-β signaling pathway, and MAPK signaling pathway, thereby providing insights into the mechanism of the effects of HJT on GC.

**Conclusion:** This study revealed the potential bioactive components and molecular mechanisms of HJT, which may be useful for the treatment of GC, and provided insights into the development of new drugs for GC.

## Graphical Abstract

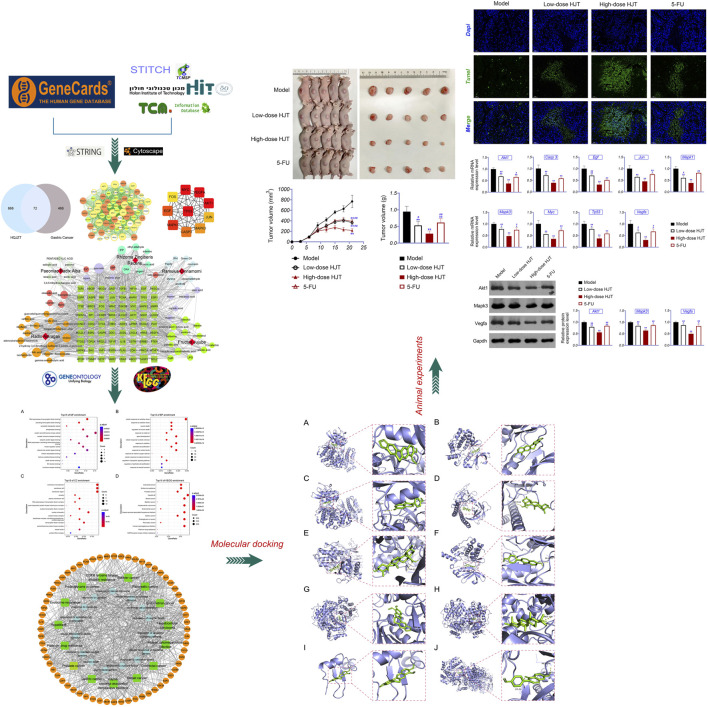

## Introduction

Gastric cancer (GC) is one of the most common malignant tumors of the digestive tract and the second leading cause of cancer-related deaths worldwide, with 984,000 new cases and 841,000 deaths annually (Fitzmaurice et al., 2015). GC is caused by many factors, including diet, genetic susceptibility, and smoking. However, most cases of GC (89%) are attributed to *Helicobacter pylori* infection (Compare et al., 2010; Plummer et al., 2016). Currently, the treatment of GC is dependent on the disease stage, with only 30% of patients considered eligible for surgery. As most patients with GC are in the middle and late stages at the time of diagnosis, the 5-years survival rate is less than 30% (Sasako, 2008). Therefore, identifying an alternative or adjuvant treatment with few side effects and high efficacy is of great clinical significance and practical value.

Chinese medicines have garnered increasing attention owing to their high efficacy, safety, and low risk of adverse effects. Huangqi Jianzhong Tang (HJT) was described in the Synopsis of the Golden Chamber, written by Zhongjing Zhang during the Eastern Han Dynasty (A.D. 150–219). HJT consists of seven herbs, including *Radix Astragali* (Huangqi)*, Paeoniae Radix Alba* (Baishao), *Ramulus Cinnamomi* (Guizhi), *Rhizoma Zingiberis Recens* (Shengjiang), *Radix Glycyrrhizae* (Gancao), *Fructus Jujube* (Dazao), and *Saccharum Granorum* (Yitang). Among them, Yitang and Dazao can be used to treat qi deficiency in the spleen and stomach; Yitang can inhibit the growth and reproduction of microorganisms in the preparation; Baishao and Guizhi can relieve pain, sedation, and convulsions; Shengjiang can reduce damage to the gastric mucosa caused by gastric acid and pepsin; Huangqi can replenish qi and solidify the surface, support toxins, and expel pus; and Gancao can block the effect of carcinogens on tumor growth (Cheng et al., 2016; Gu et al., 2017). HJT is a well-known formula used in the clinical treatment of chronic atrophic gastritis (Liu et al., 2020), acute myocardial infarction (Bao et al., 2020), peptic ulcers, inflammatory bowel disease, autonomic dystonia, chronic hepatitis, and chronic nephritis (Chang and Luo, 1995). Although HJT is widely used to treat GC (Hua, 2006; Wang et al., 2016; Qu, 2020), its molecular mechanism is unclear.

Network pharmacology is a discipline in which the network of biological systems is analyzed and specific signal nodes are selected for multitarget drug molecular design. Network pharmacology can be used to predict the correlation between small molecules and genes, proteins, metabolites, and other targets and networks; construct a “drug-gene-target-disease” network of action; and comprehensively and systematically characterize the intervention and impact of drugs on diseases (Huang et al., 2019). Molecular docking is a theoretical approach used to study the interaction and recognition of protein receptors with small-molecule ligands, and can predict binding modes and affinity strengths (Vakser, 2014; Xue et al., 2015; Liu et al., 2021). In general, combining network pharmacology and molecular docking in Chinese medicine research can enable screening and mechanistic exploration of active compounds.

In this study, we screened the active components of HJT and explored the underlying molecular mechanism of HJT in GC based on network pharmacology. Further, we used molecular docking and animal experiments to validate the effects of HJT against GC, ultimately providing a theoretical basis for its clinical application as a treatment for GC.

## Methods

### Collection of the Therapeutic Targets for GC

The therapeutic targets for GC were obtained from the Malacard (https://www.malacards.org/) (Rappaport et al., 2013) module in GeneCards (Rappaport et al., 2017) (https://www.genecards.org/) using “*gastric cancer*” as the keyword for the search (Retrieval deadline: 2020.10).

### Collection of the Bioactive Ingredients and Therapeutic Targets of HJT

The active ingredients of HJT were searched in the Traditional Chinese Medicine Integrated Database (TCMID, http://www.megabionet.org/tcmid/) (Huang et al., 2018), Traditional Chinese Medicine Systems Pharmacology Database and Analysis Platform (TCMSP, https://old.tcmsp-e.com/tcmsp.php) (Ru et al., 2014), and Herb Ingredients’ Targets (HIT, http://hit2.badd-cao.net/) (Ye et al., 2011) databases. A total of 331 active compounds were obtained following the exclusion of compounds that lacked target information. Additionally, the Search Tool for Interacting Chemicals (STITCH, http://stitch.embl.de) (Szklarczyk et al., 2016) database was used to retrieve the compound targets. A total of 8,322 targets were obtained by selecting targets with compound-target association scores greater than 400 in the STITCH database and normalizing the target information using the Gene module in the NCBI database.

Drug-likeness evaluation of chemical components was mainly used to evaluate the absorption, distribution, metabolism, and excretion properties of compounds. An effective quantitative estimate of the drug-likeness evaluation index described previously (Bickerton et al., 2012; Liang et al., 2014; Yang et al., 2018) was used to identify pharmaceutically active compounds in HJT. By referring to DrugBank drugs with a quantitative estimate of drug-likeness value greater than 0.2, 292 compounds satisfying the drug-likeness evaluation were obtained.

Finally, by using a binomial statistical model (Liang et al., 2014; Yang et al., 2018) to screen the core targets of HJT, 738 major targets and 257 major compounds were identified.

### Protein-Protein Interaction Network Construction

HJT-related and GC-related targets were subjected to Venny analysis to identify the cross-targets, and ultimately reveal the corresponding active compounds. The cross-targets were then uploaded to the Search Tool for the Retrieval of Interacting Genes (STRING) Database (https://string-db.org). The PPI network was analyzed using CytoHubba, a plug-in of Cytoscape (v3.8.0), and screened with a median value of degrees (Sun et al., 2019).

In the network, the size, form, and color of a node represent the value of the degree. Of note, the value of the degree increases as the node becomes more important.

### GO and KEGG Pathway Enrichment Analyses

The RGUI and ClusterProfiler packages in R (4.0) (http://bioconductor.org/packages/release/bioc/html/clusterProfiler.html) (Yu et al., 2012) were used to perform the Gene Ontology (GO) and Kyoto Encyclopedia of Genes and Genomes (KEGG) pathway enrichment analyses, with adjusted *p*-values of <0.01 using the Bonferroni algorithm (Retrieval deadline: 2020.10).

### Molecular Docking Analysis

The chemical structure of the active compound was obtained from the Zinc [23] database (http://zinc15.docking.org) and then imported into AutoDockTools-1.5.6 software to add polar hydrogen, distribute the charge, and set the rotatable bond. The resulting structure was saved in “pdbqt” format. The 3D structure of the protein was retrieved from the Protein Data Bank (PDB, http://www.rcsb.org/) and then inputted into PyMOL (v2.3.0) to remove water molecules, co-crystallized ligands, and ions. The AutoDockTools-1.5.6 software was used to add polar hydrogen and distribute the charge; the resulting structure was saved in “pdbqt” format. Molecular docking analysis was performed using AutoDock Vinna (v1.1.2) software. Affinity reflects the score for molecular docking; a lower score indicates stronger binding affinity. In this study, an affinity of less than −7 kcal/mol was considered to indicate strong binding activity.

### HJT Preparation

HJT herbal drink (448 g, Tongrentang, Beijing, China) was soaked in 1,000 ml of distilled water for 30 min and condensed and refluxed for 60 min. The filtrate was then collected and concentrated to 170 ml under reduced pressure in a rotary evaporator at 45°C to obtain a final HJT concentration of 2.64 g/ml. Finally, the HJT solution was diluted with water to concentrations of 0.66 and 1.32 g/ml.

### High Performance Liquid Chromatography Analysis

HPLC analysis were performed using the Agilent 1260 HPLC system (Agilent Technologies, Santa Clara, CA, United States) equipped with a Zorbax C_18_ column (250 mm ⅹ 4.6 mm, 5 μm). The following gradient elution of methanol-water was performed: 0–10 min, 30% methanol; 10–20 min, 40% methanol; 20–30 min, 50% methanol; 30–40 min, 65% methanol; 40–50 min, 80% methanol; 50–60 min, 95% methanol. The column temperature was maintained at 25°C, the detection wavelength was 250 nm, the flow rate was 1.0 ml/min, and the injection volume was 10 μL.

### Cell Culture and Animal Model

Human GC cells (MGC-803) were purchased from the Chinese Academy of Sciences Shanghai Cell Bank (Shanghai, China) and cultured in RPMI-1640 medium supplemented with 10% fetal bovine serum (Gibco, Grand Island, NY, United States) at 37°C with 5% CO_2_.

Male BALB/c nude mice (6-week-old) were housed in a controlled environment under a 12-h light/dark cycle with free access to water and food. MGC-803 cells (2 × 10^6^) were inoculated subcutaneously into the right side of each mouse, and tumor size was observed daily. When the tumor reached approximately 3 mm × 3 mm, mice were randomly divided into four groups (*n* = 5 in each group). Mice in the HJT group were administered 0.66 g/ml (low-dose) and 1.32 g/ml (high-dose) HJT (0.5 ml/20 g) via gavage each day; mice in the model group were administered equal amounts of saline via gavage; and mice in the positive control group were administered an intraperitoneal injection of 5-fluorouracil (5-FU, 20 mg/kg, Sigma-Aldrich, St. Louis, MO, United States) every 2 days. The tumor volume was calculated every 3 days (formula: A × B^2^/2, where A is the long diameter and B is the short diameter); only one measurement was performed. After 15 days, mice were killed, and the tumors were isolated and weighed. All animal experiments were approved by the Experimental Animal Management and Ethics Committee of Xiamen University.

### TUNEL Staining

To detect cellular apoptosis, the tumor tissue sections were deparaffinized and rehydrated. The sections were then incubated with 20 μg/ml proteinase K for 30 min at 37°C and TdT reaction mix for 60 min at 37°C. After three rounds of washing with phosphate-buffered saline, the sections were stained with 4′,6-diamidino-2-phenylindole staining solution for 10 min at room temperature in the dark. Finally, the sections were observed using a fluorescence microscope (Olympus, Tokyo, Japan).

### 2.10 Quantitative Real-Time PCR

Total RNA was extracted from the tumor tissues using TRIzol reagent (Invitrogen, Carlsbad, CA, United States) and reverse-transcribed into cDNA using cDNA reverse transcription kits (TransGen Biotech Co., Ltd., Beijing, China). SYBR Green PCR Master Mix (Takara, Shiga, Japan) was used to amplify the samples on the MX3000P Real-Time QPCR System (Agilent Technologies, Santa Clara, CA, United States). The following cycling conditions were employed for PCR: 95°C for 3 min, 40 cycles at 95°C for 12 s, and 60°C for 40 s. Relative mRNA expression was detected using the 2^−ΔΔCt^ method, with Gapdh as the reference gene. The primers used in the qPCR assays are listed in Supplementary Table S1.

### Western Blot Analysis

Tumor tissue homogenates were prepared in lysis buffer (Beyotime, Shanghai, China). The proteins in the homogenates were separated via sodium dodecyl sulfate-polyacrylamide gel electrophoresis and transferred onto polyvinylidene fluoride membranes. After blocking with 5% skim milk for 90 min, the membranes were incubated with anti-Mapk3 (ab32537), anti-Akt1 (ab227385), anti-Vegfa (ab214424), and anti-Gapdh (ab9485) (1:1000, Abcam, Cambridge, United Kingdom) overnight at 4°C, followed by the corresponding secondary antibody (1:1000, ab6721, Abcam) for 1 h at room temperature. The blots were detected using an enhanced chemiluminescence kit and GeneGnome XRQ (Gene Company, China). The gray values of the bands were quantified using ImageJ software (NIH, Bethesda, MD, United States).

### Statistical Analysis

GO and KEGG pathway enrichment analyses were performed with adjusted *p*-values of <0.01 based on the Bonferroni algorithm.

All data are expressed as mean ± SD and were analyzed using GraphPad Prism 7.0 software (GraphPad, Inc., La Jolla, CA, United States). One-way analysis of variance followed by Tukey’s test was used for multiple comparisons among groups. A *p* value <0.05 indicated statistical significance.

## Results

### Target Identification

**FIGURE 1:**
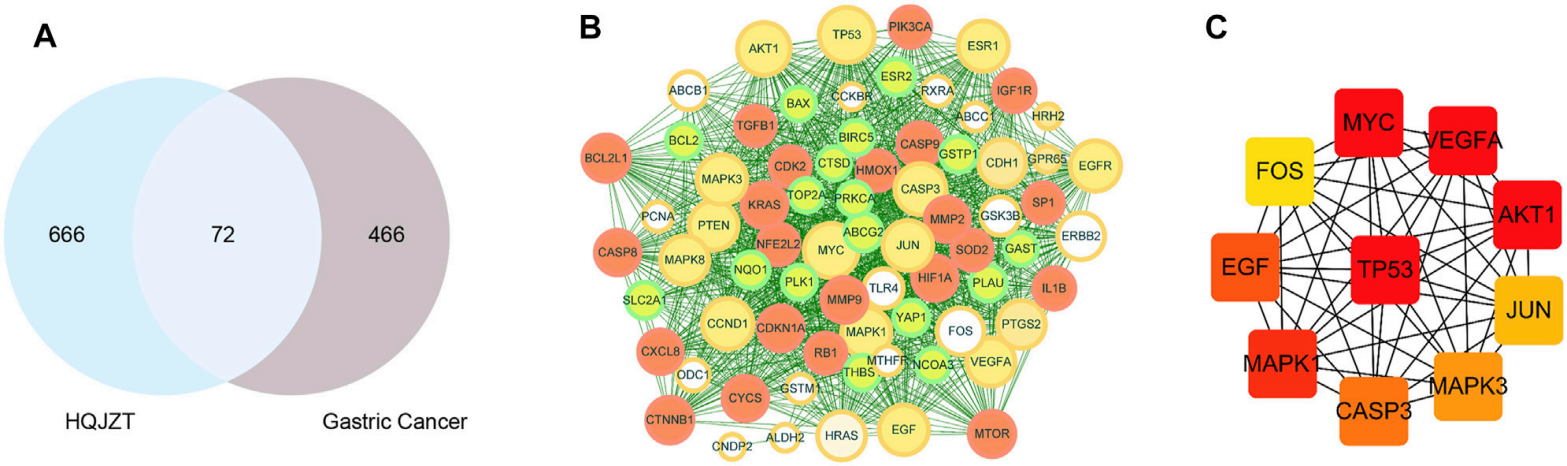
**(A)** Venn diagram of the targets of Huangqi Jianzhong Tang (HJT) for the treatment of gastric cancer (GC). A total of 738 HJT-related targets and 538 GC-related targets were identified, and a total of 72 cross-targets were obtained using Venny. **(B)** The PPI network of the 72 cross-targets were constructed using Cytoscape. The size of nodes represents their degree values. **(C)** The top 10 hub genes were screened from **(B)** using CytoHubba.

**FIGURE 2:**
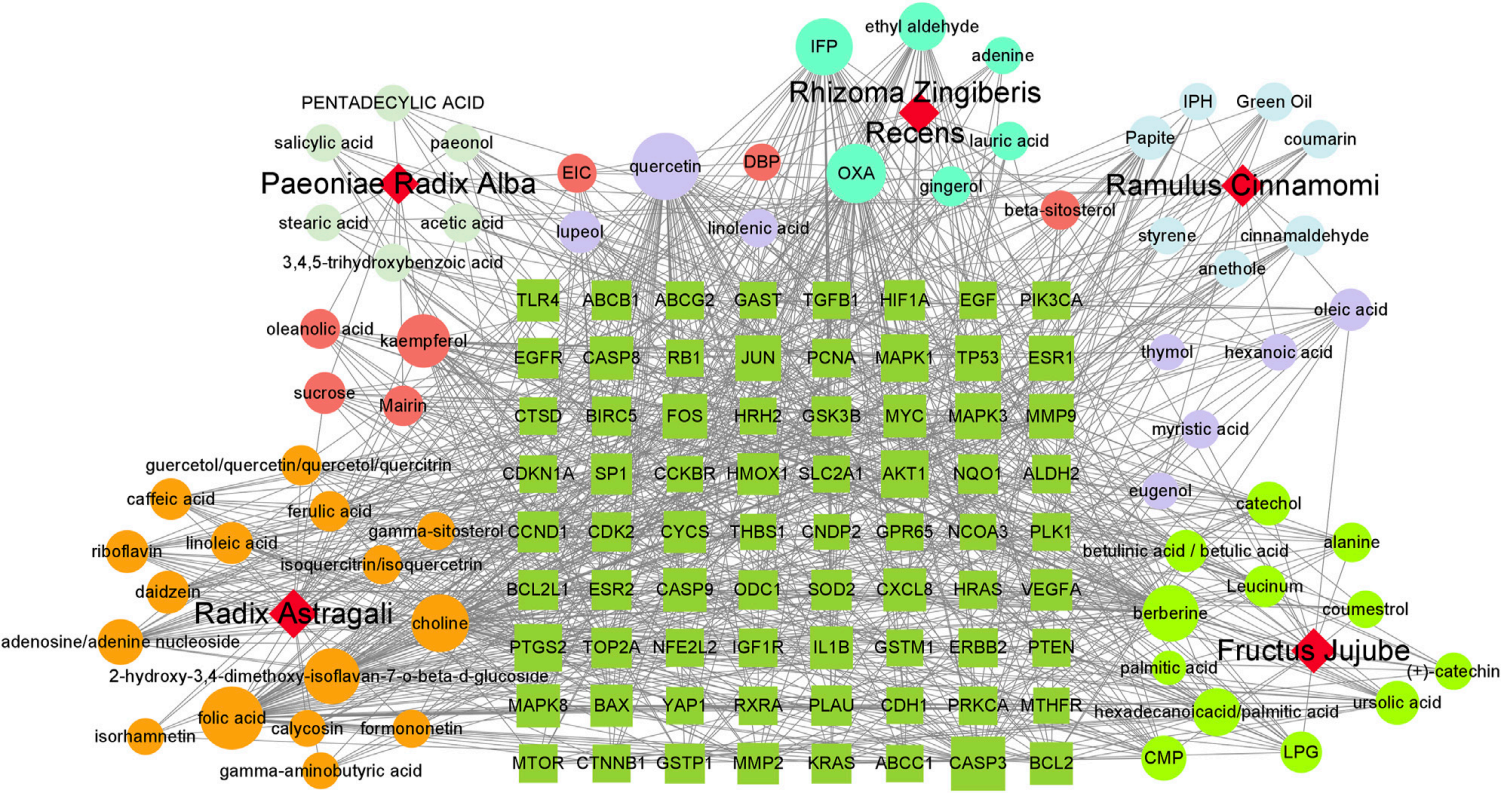
Network of the HJT herb-component-target. The dark green square represents the key target of the compound for gastric cancer, circle represents the compound, and different colors represent the compounds contained in different traditional Chinese medicines; pink and lavender represent compounds found in many types of traditional Chinese medicine; red quadrilateral represents different traditional Chinese medicines.

A total of 538 GC targets were obtained from the GeneCards database. Further, 72 cross-targets were obtained between HJT and GC using Venny (Figure 1A; Table 1). Cytoscape (v3.8.0) was used to construct the PPI network, which contained 72 nodes and 1,194 edges with an average degree value of 33.2 (Figure 1B). Ten hub genes (*FOS*, *EGF*, *MAPK1*, *CASP3*, *MAPK3*, *JUN*, *AKT1*, *VEGFA*, *MYC*, and *TP53*) were selected using CytoHubba software with high degrees. Thereafter, a hub gene network of 10 nodes and 45 edges was constructed using the genes listed above (Figure 1C). A total of 89 bioactive compounds that met the criteria for drug-likeness screening and had GC treatment targets were obtained using the 72 cross-targets. A total of 63 bioactive compounds were selected from 89 bioactive compounds based on a frequency ≥10; further details are provided in Table 2. Cytoscape was used to construct a visual HJT herb-component-target network, which consisted of 139 nodes and 942 edges. Quercetin, folic acid, oxaliplatin, choline, IFP, and berberine had a high degree in this study, suggesting that they play important roles in the effects of HJT on GC (Figure 2).

**TABLE 1:** The target genes of Huangqi Jianzhong Tang.

ID	Name	ID	Name	ID	Name	ID	Name
3725	JUN	55748	CNDP2	3265	HRAS	5290	PIK3CA
5243	ABCB1	1499	CTNNB1	3274	HRH2	5328	PLAU
4363	ABCC1	1509	CTSD	3480	IGF1R	5347	PLK1
9429	ABCG2	3576	CXCL8	3553	IL1B	5578	PRKCA
207	AKT1	54205	CYCS	3845	KRAS	5728	PTEN
217	ALDH2	1950	EGF	5594	MAPK1	5743	PTGS2
581	BAX	1956	EGFR	5595	MAPK3	5925	RB1
596	BCL2	2064	ERBB2	5599	MAPK8	6256	RXRA
598	BCL2L1	2099	ESR1	4313	MMP2	6513	SLC2A1
332	BIRC5	2100	ESR2	4318	MMP9	6648	SOD2
836	CASP3	2353	FOS	4524	MTHFR	6667	SP1
841	CASP8	2520	GAST	2475	MTOR	7040	TGFB1
842	CASP9	8477	GPR65	4609	MYC	7057	THBS1
887	CCKBR	2932	GSK3B	8202	NCOA3	7099	TLR4
595	CCND1	2944	GSTM1	4780	NFE2L2	7153	TOP2A
999	CDH1	2950	GSTP1	1728	NQO1	7157	TP53
1017	CDK2	3091	HIF1A	4953	ODC1	7422	VEGFA
1026	CDKN1A	3162	HMOX1	5111	PCNA	10413	YAP1

**TABLE 2:** Bioactive compounds of Huangqi Jianzhong Tang.

Chemical	QED	Chemical	QED
Palmitic acid	0.3653	Anethole	0.6262
Quercetin	0.5064	Isorhamnetin	0.6678
Kaempferol	0.6372	Acetic acid	0.4199
OXA	0.4049	Coumarin	0.4124
Folic acid	0.2979	Ferulic acid	0.7180
IFP	0.3920	Oleanolic acid	0.5678
Choline	0.3405	Coumestrol	0.4848
Berberine	0.6633	Green Oil	0.4608
Mairin	0.4635	Hexanoic acid	0.5687
Lupeol	0.4329	Oleanolic acid	0.4460
Hexadecanoicacid	0.4133	Stearic acid	0.3017
Oleic acid	0.2030	Styrene	0.5128
2-hydroxy-3,4-dimethoxy-isoflavan-7-o-beta-d-glucoside	0.6633	formononetin	0.8529
Catechol	0.4946	isoquercitrin/isoquercetrin	0.2745
EIC	0.2944	eugenol	0.6955
CMP	0.5211	gamma-sitosterol	0.4354
Linolenic acid	0.3326	gingerol	0.6465
Sucrose	0.2411	3,4,5-trihydroxybenzoic acid	0.4656
Cinnamaldehyde	0.4437	lauric acid	0.3925
Papite	0.3158	paeonol	0.5478
Beta-sitosterol	0.4354	(+)-catechin	0.5139
Daidzein	0.8195	calycosin	0.8850
Adenosine	0.4953	gamma-aminobutyric acid	0.3980
Betulinic acid/betulic acid	0.5913	Guercetol	0.5064
Linoleic acid	0.2944	IPH	0.5172
Leucinum	0.4686	thymol	0.6510
LPG	0.3562	Salicylic acid	0.6129
Riboflavin	0.4674	Adenine	0.5125
DBP	0.4752	Alanine	0.3562
Ethyl aldehyde	0.3445	Myristic acid	0.4490
Caffeic acid	0.4750	Pentadecylic acid	0.4059
Ursolic acid	0.4433		

### GO and KEGG Pathway Enrichment Analyses of HJT

**FIGURE 3:**
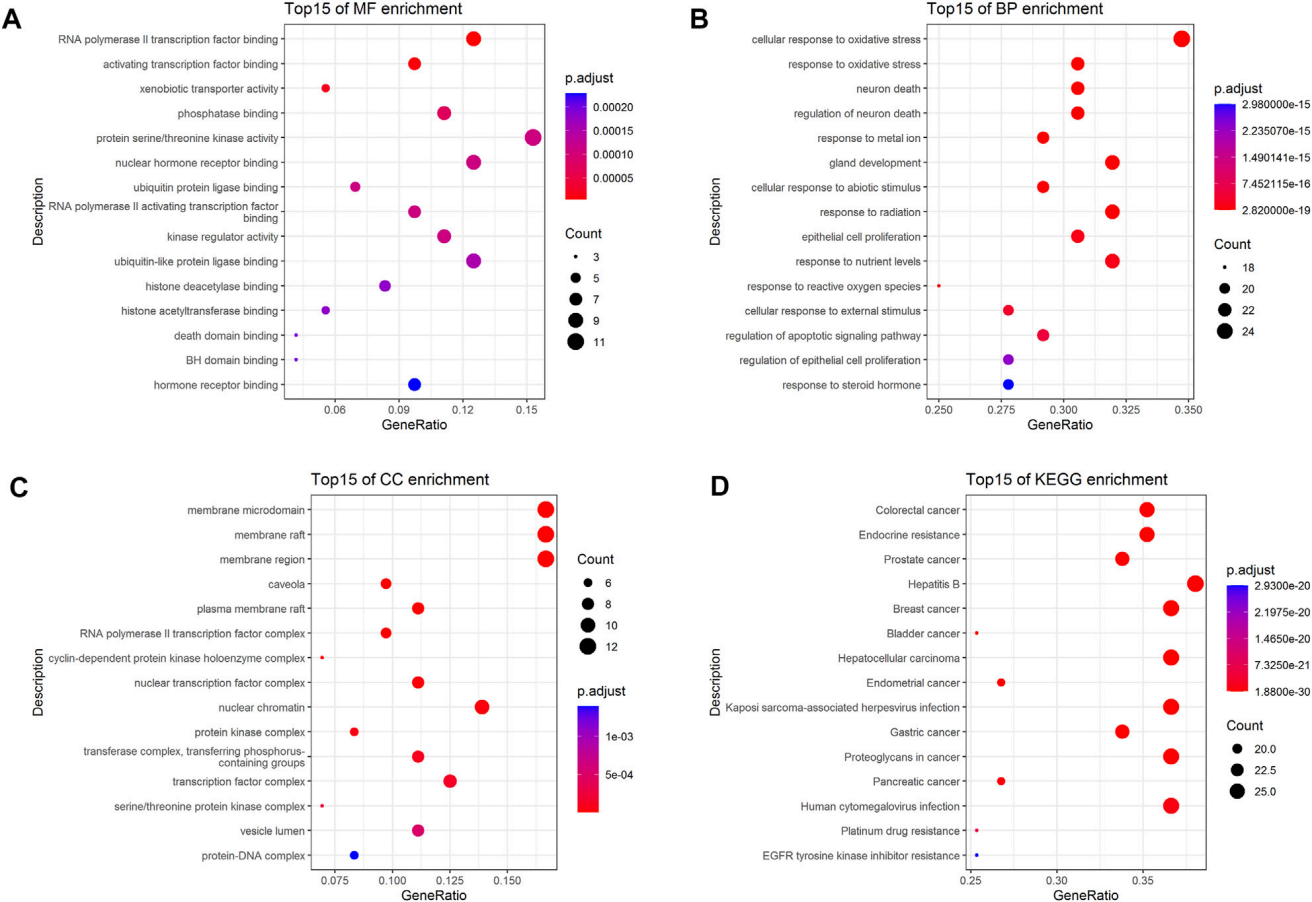
GO and KEGG enrichment analysis. **(A)** Top 15 significantly enriched terms in molecular function (MF); **(B)** Top 15 significantly enriched terms in biological process (BP); **(C)** Top 15 significantly enriched terms in cellular component (CC); **(D)** Top 15 significantly enriched terms in the KEGG pathway. Gene ratio = count/set size.

**FIGURE 4:**
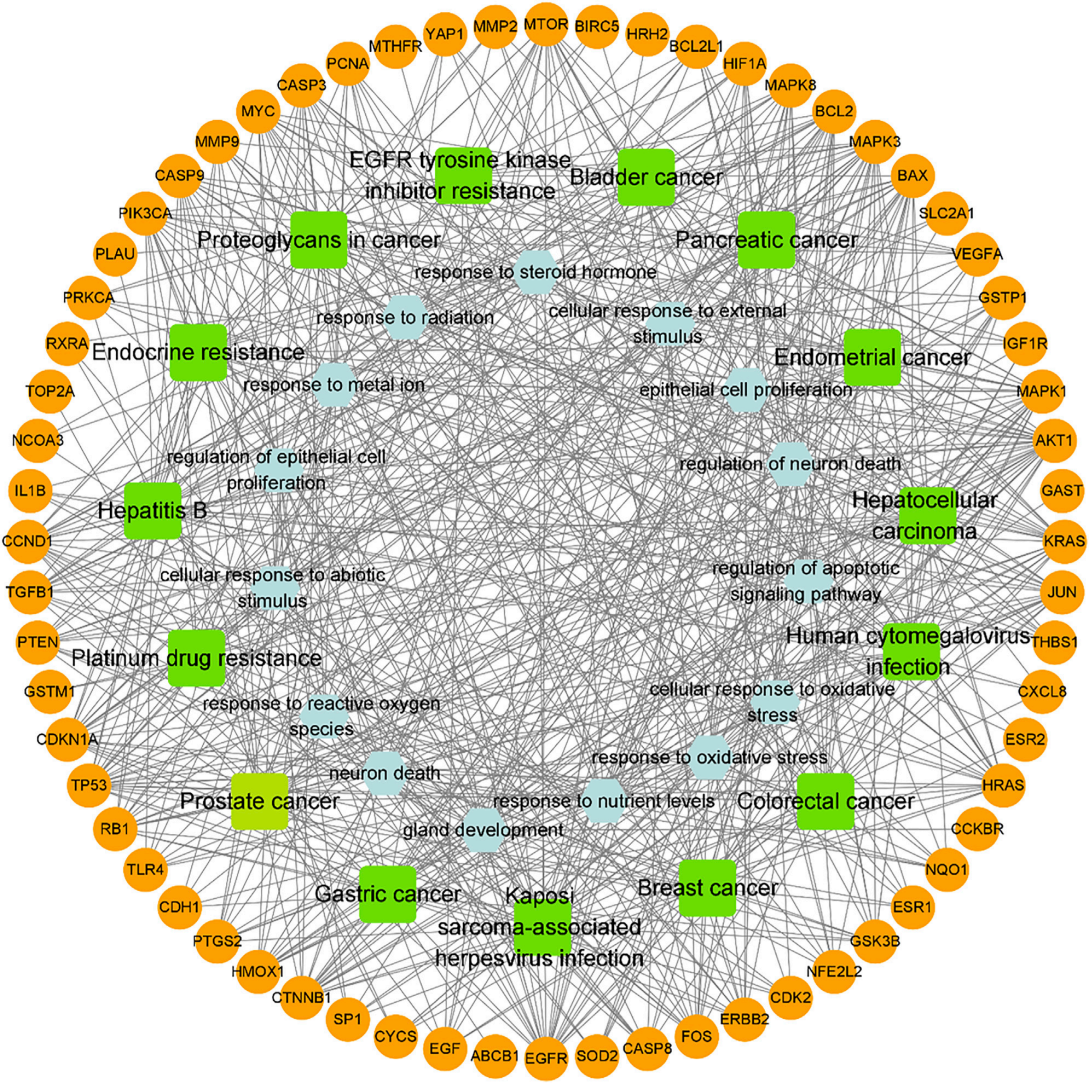
Network of the target-biological process pathway. The orange circle represents the target of the compound acting on the disease, green square represents the co-associated pathway, and light blue hexagon represents the top 15 biological process-related terms.

To further investigate the 72 cross-targets of the HJT bioactive compounds, GO enrichment analysis was performed to elucidate the biological process (BP), molecular function (MF), and cellular component (CC) terms. A total of 41 MFs, 1,337 BPs, and 28 CCs were found to be enriched (*p* < 0.01). The GO analysis results for the top 15 markedly enriched MF, BP, and CC terms are shown in Figures 3A–C. KEGG pathway enrichment analysis revealed that the 72 cross-targets of the HJT bioactive compounds were enriched in 140 pathways (*p* < 0.01). The top 15 enriched KEGG pathways for the HJT bioactive compounds are shown in Figure 3D. Furthermore, to illustrate the correlation between cross-targets and the top 15 BP-related terms and top 15 KEGG pathways, a target-BP-pathway network diagram was constructed (Figure 4).

### Determination of the Active Compounds in HJT

The main active ingredients in HJT were analyzed by HPLC. By comparing HJT to the standard, formononetin was identified as the dominant compound, followed by cinnamaldehyde, gingerol, ursolic acid, anethole, and berberine (Supplementary Figure S1).

### Docking Stimulation Verification

**FIGURE 5:**
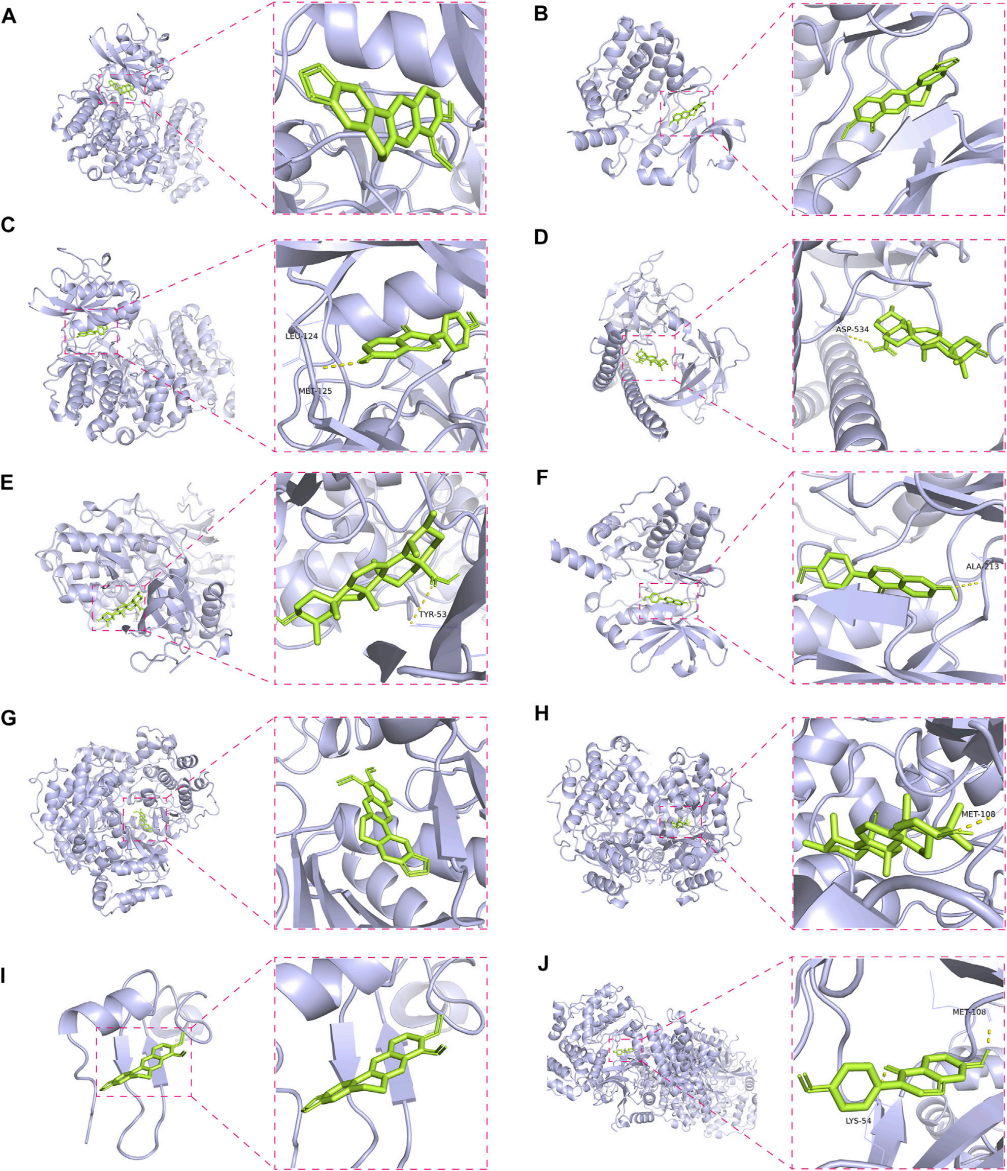
Molecular docking of the hub targets with bioactive compounds. **(A)** Binding poses of berberine complexed with MAPK, affinity = −9.2 kcal/mol; **(B)** Binding poses of berberine complexed with MYC, affinity = −8.8 kcal/mol; **(C)** Binding poses of formononetin complexed with MAPK3, affinity = −8.7 kcal/mol; **(D)** Binding poses of ursolic acid complexed with JUN, affinity = −8.1 kcal/mol; **(E)** Binding poses of ursolic acid complexed with MAPK3, affinity = −8.1 kcal/mol; **(F)** Binding poses of formononetin complexed with MYC, affinity = -8 kcal/mol; **(G)** Binding poses of berberine complexed with MAPK1, affinity = −7.8 kcal/mol; **(H)** Binding poses of ursolic acid complexed with MAPK1, affinity = −7.7 kcal/mol; **(I)** Binding poses of berberine complexed with EGF, affinity = −7.4 kcal/mol; **(J)** Binding poses of formononetin complexed with MAPK1, affinity = −7.3 kcal/mol.

To validate the candidate GC targets in HJT, molecular docking was used to identify the binding ability between the bioactive components (berberine, formononetin, ursolic acid, gingerol, anethole, and cinnamaldehyde). The corresponding 2D-chemical structures of HJT and the hub targets (*MYC*, *VEGFA*, *AKT1*, *JUN*, *MAPK3*, *CASP3*, *MAPK1*, *EGF*, and *TP53*), generated using Zinc software, are presented in Table 3. As shown in Table 4, 41 pairs of docking results were obtained, and berberine had a strong binding activity to MAPK3 (affinity = -9.2 kcal/mol). The top 10 affinities of the combination of bioactive components and hub targets are shown in Figure 5.

**TABLE 3:** The chemical structure of Huangqi Jianzhong Tang bioactive compounds.

Synonyms	Molecular formula	2D structure
berberine	C20H18NO4+	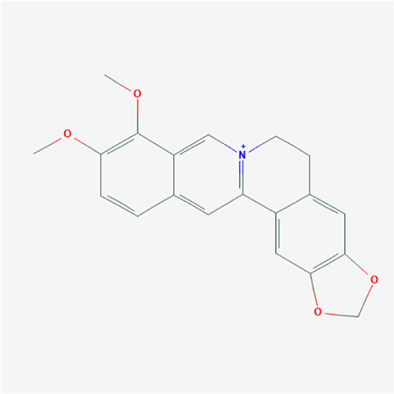
anethole	C10H12O	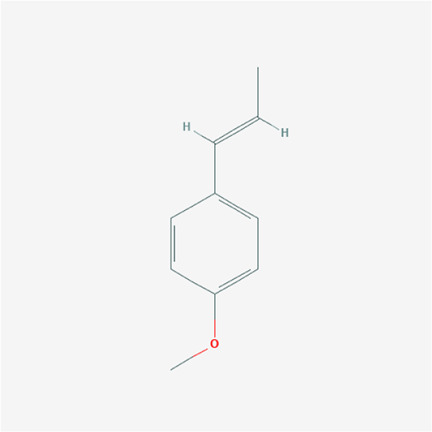
formononetin	C16H12O4	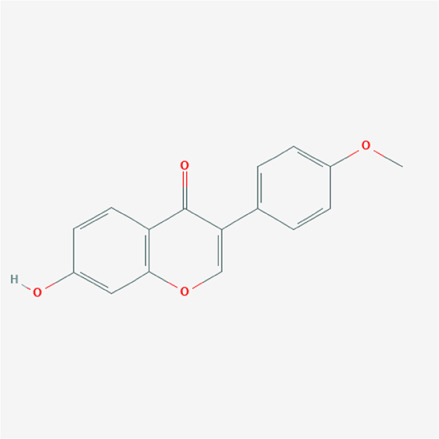
cinnamaldehyde	C9H8O	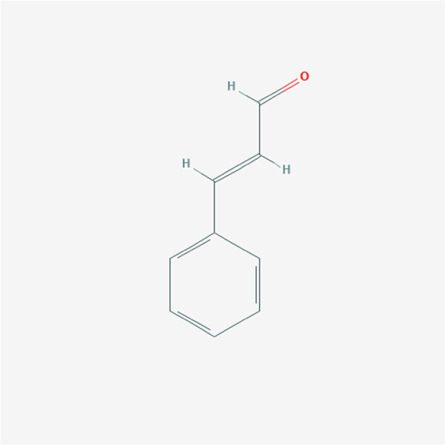
gingerol	C17H26O4	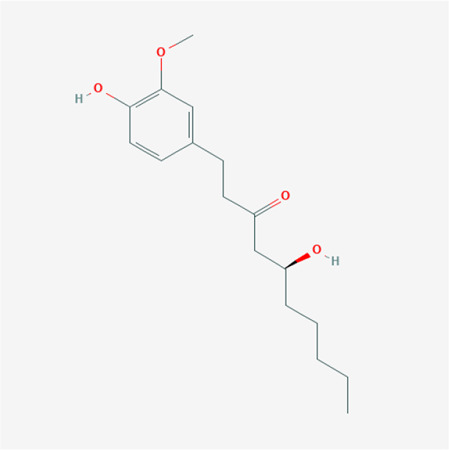
ursolic acid	C30H48O3	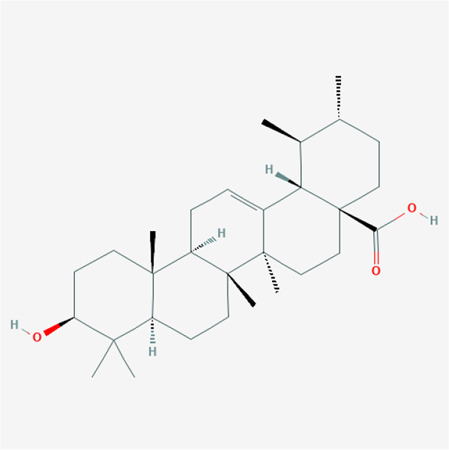

**TABLE 4:** The result of molecular docking.

Chem	PDB	Gene	Best affinity
Berberine	4qtb	MAPK3	−9.2
Berberine	5g1x	MYC	−8.8
Formononetin	4qtb	MAPK3	−8.7
Ursolic acid	1s9k	JUN	−8.1
Ursolic acid	4qtb	MAPK3	−8.1
Formononetin	5g1x	MYC	−8
Berberine	4iz5	MAPK1	−7.8
Ursolic acid	4iz5	MAPK1	−7.7
Berberine	2kv4	EGF	−7.4
Formononetin	4iz5	MAPK1	−7.3
Formononetin	2kv4	EGF	−7.3
Berberine	1s9k	JUN	−7.2
Gingerol	4qtb	MAPK3	−6.8
Ursolic acid	2kv4	EGF	−6.7
Ursolic acid	3q05	TP53	−6.7
Gingerol	5g1x	MYC	−6.6
Formononetin	1s9k	JUN	−6.5
Berberine	2xyp	CASP3	−6.1
Ursolic acid	1unq	AKT1	−6.1
Ursolic acid	5g1x	MYC	−6
Anethole	5g1x	MYC	−5.9
Anethole	4qtb	MAPK3	−5.9
Gingerol	4iz5	MAPK1	−5.8
Berberine	3q05	TP53	−5.7
Cinnamaldehyde	4qtb	MAPK3	−5.7
Gingerol	1s9k	JUN	−5.7
Formononetin	2xyp	CASP3	−5.6
Formononetin	3q05	TP53	−5.6
Cinnamaldehyde	5g1x	MYC	−5.6
Anethole	2kv4	EGF	−5.5
Gingerol	2kv4	EGF	−5.5
Berberine	1unq	AKT1	−5.4
Cinnamaldehyde	1s9k	JUN	−5.4
Ursolic acid	4gln	VEGFA	−5.4
Ursolic acid	2xyp	CASP3	−5.4
Formononetin	1unq	AKT1	−5.3
Cinnamaldehyde	2kv4	EGF	−5.3
Berberine	4gln	VEGFA	−5.2
Anethole	1s9k	JUN	−5.2
Formononetin	4gln	VEGFA	−5.2
Gingerol	2xyp	CASP3	−5.1

### Experimental Verification

**FIGURE 6:**
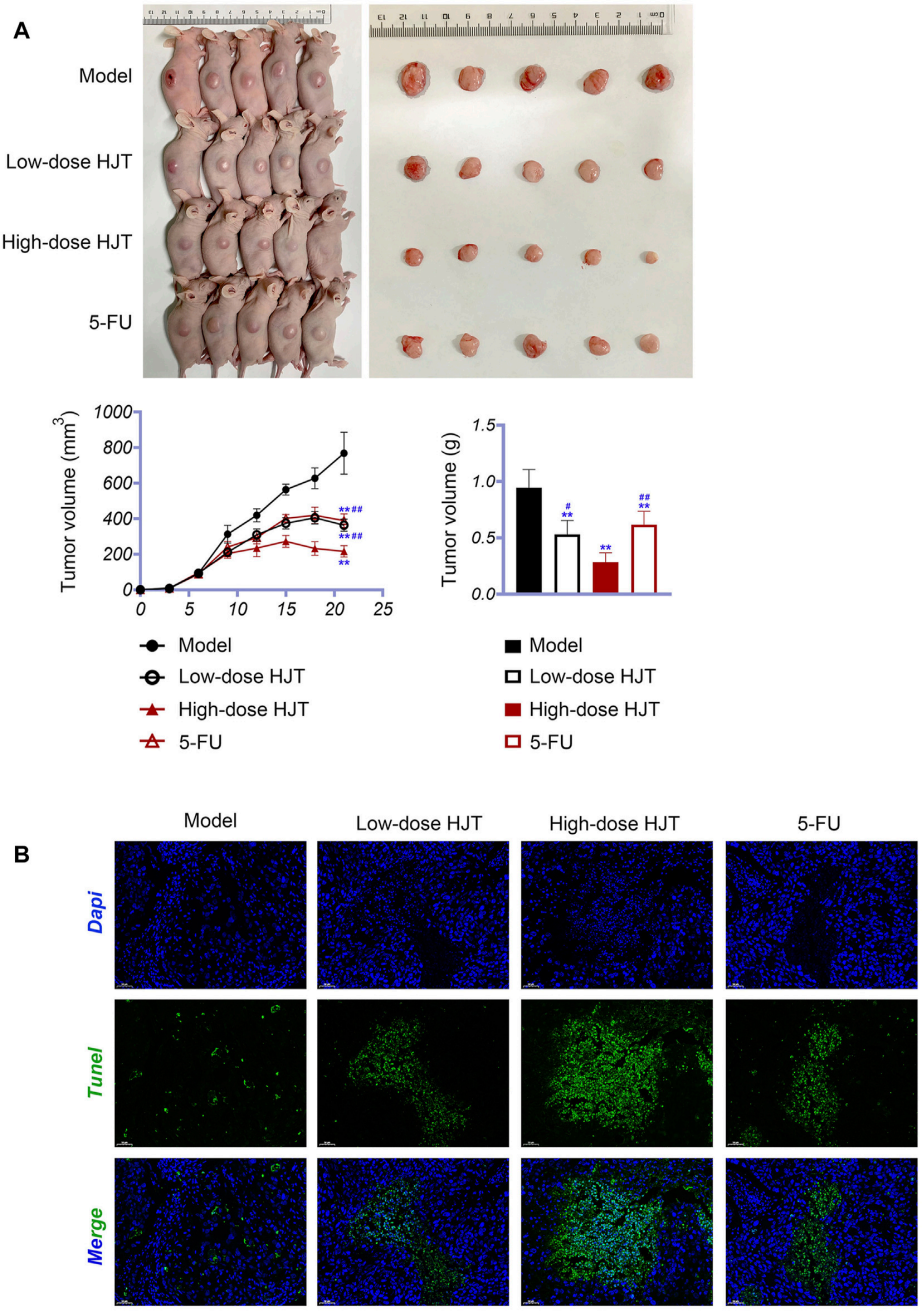
HJT suppressed tumor growth. **(A)** Human GC cells (MGC-803) were inoculated subcutaneously on the right side of nude mice. Thereafter, tumors were observed in the control, low-dose HJT (0.66 g/ml gavage), high-dose HJT (1.32 g/ml gavage), and positive control (5-FU, 20 mg/kg *ip*.) groups. The tumor volume was calculated every 3 days, with only one measurement performed. After 15 days, mice were killed, and the tumors were isolated and weighed. **(B)** The apoptosis of tumor tissues in all four groups was detected using the TUNEL assay, scale bar = 50 μm **p < 0.01 *vs*. Control group. #*p* < 0.05 and ##*p* < 0.01 *vs*. High-dose HJT group.

Nude mice xenografted subcutaneously with MGC-803 cells were used to investigate the potential therapeutic effects of HJT. Compared to treatment with the model compound, HJT and 5-FU significantly decreased the tumor size and weight (*p* < 0.01). Moreover, compared with low-dose HJT and 5-FU, high-dose HJT most effectively inhibited tumor growth (*p* < 0.01) (Figure 6A).

The apoptotic effects of HJT on tumor tissues were determined using a TUNEL assay. As shown in Figure 6B, treatment with high-dose HJT led to a higher apoptosis rate of tumor tissues.

**FIGURE 7:**
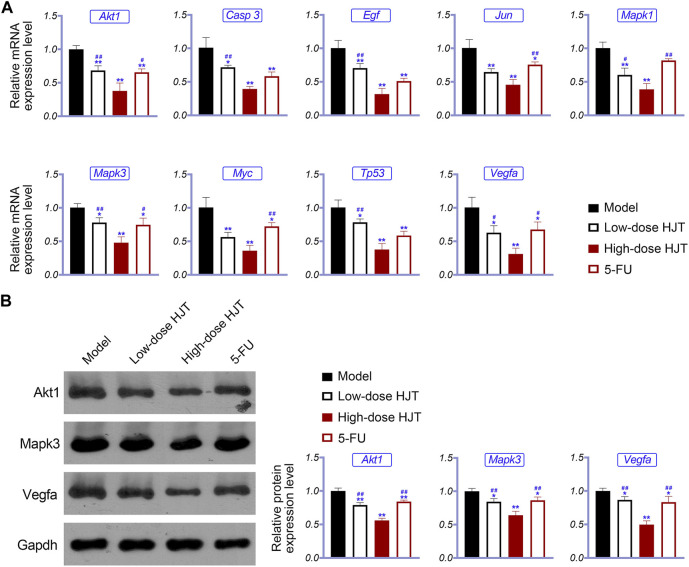
Expression of hub genes in the tumor tissue of the control, low-dose HJT (0.66 g/ml gavage), high-dose HJT (1.32 g/ml gavage), and positive control (5-FU, 20 mg/kg ip.) groups. qRT-PCR was used to determine the relative mRNA expression of Akt1, Casp3, Egf, Jun, Mapk1, Mapk3, Myc, Tp53, and Vegfa **(A)**, while western blotting was carried out to detect the relative protein expression of Akt1, Mapk3, and Vegfa **(B)**.

To further study the anti-GC effects of HJT, we selected the hub genes identified in the network pharmacology analysis as potential targets of HJT for qRT-PCR and western blot analysis. Based on the qRT-PCR results, the expression levels of Akt1, Casp3, Egf, Jun, Mapk1, Myc, Tp53, Mapk3, and Vegfa in the HJT and 5-FU treatment groups were notably downregulated compared to those in the model group, (*p* < 0.05, *p* < 0.01). Further, based on western blotting, the protein expression levels of Mapk3, Akt1, and Vegfa in the treatment group were significantly decreased compared to those in the model group (*p* < 0.05, *p* < 0.01) (Figure 7).

## Discussion

In traditional Chinese medicine, GC is characterized by symptoms, such as epigastric pain, nausea, and choking hiccups (Zhang and Zhang, 2012). The receptive, ripening, and digestive functions of the stomach depend on transformation of the spleen and liver; therefore, stomach disease is closely related to the spleen and liver. Clinically, GC of the spleen-stomach deficiency-cold type is common (Zhu et al., 2012). According to prior studies, HJT can invigorate and warm the spleen and relieve pain in the stomach (Zheng et al., 2010). However, the mechanism of the effects of HJT on GC remains unclear.

In this study, 63 bioactive components were identified in HJT, with folic acid, choline, kaempferol, quercetin, formononetin, cinnamaldehyde, gingerol, ursolic acid, anethole, and berberine identified as the main components. According to previous studies, dietary folic acid can protect against certain types of cancer. Gonda et al. reported that folic acid supplementation prevented the loss of global DNA methylation, markedly reduced gastric dysplasia and mucosal inflammation, and reduced inflammation to prevent *Helicobacter*-associated GC in mice (Gonda et al., 2012). Choline is an effective inhibitor of GC cell progression, and combining Notch1 inhibitors with crocodile choline may be useful for treating GC through a mechanism related to the mitochondrial apoptosis pathway and Notch pathway (Mao et al., 2017). Kaempferol is a plant-derived flavonoid with a wide range of pharmacological activities. In a previous study, kaempferol was demonstrated to suppress proliferation and promote autophagy in human GC SNU-216 cells by inactivating the MAPK/ERK and PI3K pathways (Zhang and Ma, 2019). Quercetin is a flavonoid found in a wide variety of vegetables and fruits that can enhance the efficacy of anticancer drugs. Quercetin has been shown to inhibit cell growth; induce apoptosis, necrosis, autophagy, and anti-*Helicobacter pylori* activity; and exhibit low bioavailability (Haghi et al., 2017). Formononetin is one of the major isoflavonoid constituents isolated from Huangqi and has diverse pharmacological activities, including anticancer effects. Wang et al. revealed that formononetin exerted antitumor activity on GC *in vitro* and *in vivo* by regulating microRNA-542-5p expression (Wang and Zhao, 2021). Cinnamaldehyde is one of the most important bioactive ingredients in Guizhi. A previous study revealed that cinnamaldehyde mediates endoplasmic reticulum stress and autophagic cell death via the PERK-CHOP signaling pathway, the inhibition of G9a binding on Beclin-1 and LC3B promoter, and dissociation of Bcl-2–Beclin-1 in GC cells (Kim, 2022). 6-Gingerol is a major phenolic compound of Shengjiang that has numerous pharmacological activities, such as antioxidant and anti-inflammatory properties. According to a previous study, 6-Gingerol inhibits proliferation of GC via the STAT3 pathway *in vitro* (Li et al., 2019). Moreover, Luo et al. reported that 6-Gingerol enhances the cisplatin sensitivity of GC cells and that the mechanisms involve G1 phase arrest, migration, and invasion suppression via the PI3K/AKT signaling pathway (Luo et al., 2019). Ursolic acid, a natural compound that exists in many herbal plants, is known to obstruct GC progression through the NF-κB pathway (Chen et al., 2020), Hippo pathway (Kim et al., 2019), etc. Berberine has been demonstrated to repress human GC cell growth *in vitro* and *in vivo* by inducing cytostatic autophagy via the inhibition of MAPK/mTOR/p70S6K and Akt, ultimately providing a molecular basis for the treatment of GC (Zhang et al., 2020). Collectively, these findings suggest that HJT has the potential to treat GC through multiple compounds.

Based on KEGG pathway enrichment analysis, all targets were enriched in Wnt, PI3K-Akt, TGF-β, MAPK, and other signaling pathways. Thus, HJT may exert anticancer effects on GC by regulating cell proliferation and survival. To verify this hypothesis, we investigated the efficacy of HJT in a GC xenograft mouse model. HJT was found to significantly reduce tumor growth and promote apoptosis of tumor tissues. In particular, the expression levels of Akt1, Casp3, Egf, Jun, Mapk1, Myc, Tp53, and Vegfa were significantly reduced. PI3K and Akt are important downstream effectors of EGFR. In a previous study, PI3K-Akt signaling was demonstrated to play a central role in several cancer-related cellular processes, including growth, survival, and motility (Duan et al., 2014). According to Yan et al., activation of the EGFR/PI3K/Akt signaling pathway promotes the proliferation of GC cells (Yan et al., 2018). MYC is an important proto-oncogene with a key role in cell proliferation, differentiation, transformation, and apoptosis in GC (Gong et al., 2018). MAPK1 is a key regulatory gene in the MAPK signaling pathway, and studies have demonstrated that knockdown of MAPK1 inhibits GC cell proliferation, migration, and invasion (Guo et al., 2021). Chen et al. reported that the inhibition of the TGF-β signaling pathway significantly inhibits the migration, invasion, proliferation, and tumor growth of GC cells (Chen et al., 2019). VEGFA is a crucial angiogenic factor that can act as a potent inducer of vascular growth. The VEGF/VEGFR signaling pathway is thought to promote tumor angiogenesis, growth, invasion, and metastasis (Sharma et al., 2018), and the activation of VEGFA expression can promote GC growth and angiogenesis (Du et al., 2022; He et al., 2022).

## Conclusion

In this study, we systematically analyzed the potential effects of HJT on GC based on network pharmacology. Molecular docking revealed that each bioactive compound (formononetin, cinnamaldehyde, gingerol, ursolic acid, anethole, and berberine) of HJT had favorable binding abilities with MYC, VEGFA, AKT1, JUN, MAPK3, CASP3, MAPK1, EGF, and TP53, further implying the potential molecular mechanism of action of HJT in GC. *In vivo* experiments also revealed the changes in hub gene expression in tumor issues following HJT treatment, ultimately highlighting the ability of HJT to treat GC. Changes in hub gene expression also indicated that these hub genes may be important targets for GC therapy. Overall, our study provides a comprehensive reference for the subsequent treatment of GC. However, our study has limitations as the specific mechanism of HJT treatment for GC was not validated. Nonetheless, this task will be the focus of our subsequent studies.

## Data Availability

The original contributions presented in the study are included in the article/supplementary material. Further inquiries can be directed to the corresponding author.

## Supplementary Material

Supplementary Figure S1 HPLC chromatograms of the standard (Top) and HJT (Bottom). The peaks for the standards, from left to right, represent berberine, formononetin, gingerol, ursolic acid, cinnamaldehyde, and anethole.

Supplementary Table S1 The primer sequences used in this present study.
