# Supplementary material for: Network Pharmacology Analysis of Huangqi Jianzhong Tang Targets in Gastric Cancer
Source: Front Pharmacol. 2022 Apr 8;13:882147. doi: 10.3389/fphar.2022.882147 (PMC9024123; doi:10.3389/fphar.2022.882147)
Supplement: Supplementary file 1 [file Table1.DOCX]

| **Gene** | **Sequence (5’-3’)** |
| --- | --- |
| Myc | ATGCCCCTCAACGTGAACTTC |
|  | GTCGCAGATGAAATAGGGCTG |
| Mapk3 | TCCGCCATGAGAATGTTATAGGC |
|  | GGTGGTGTTGATAAGCAGATTGG |
| Jun | TTCCTCCAGTCCGAGAGCG |
|  | TGAGAAGGTCCGAGTTCTTGG |
| Mapk1 | GGTTGTTCCCAAATGCTGACT |
|  | CAACTTCAATCCTCTTGTGAGGG |
| Egf | AGAGCATCTCTCGGATTGACC |
|  | CCCGTTAAGGAAAACTCTTAGCA |
| Tp53 | TACTCTCCTCCCCTCAATAAGC |
|  | ACGACCTCCGTCATGTGCT |
| Casp3 | CTCGCTCTGGTACGGATGTG |
|  | TCCCATAAATGACCCCTTCATCA |
| Akt1 | ATGAACGACGTAGCCATTGTG |
|  | TTGTAGCCAATAAAGGTGCCAT |
| Vegfa | CTGCCGTCCGATTGAGACC |
|  | CCCCTCCTTGTACCACTGTC |
| Gapdh | GAAGGTCGGTGTGAACGGATTTG |
|  | CATGTAGACCATGTAGTTGAGGTCA |

**Supplementary Table S1. The primer sequences used in this present study**
