# Supplementary material for: Network Pharmacology Analysis of Huangqi Jianzhong Tang Targets in Gastric Cancer
Source: Front Pharmacol. 2022 Apr 8;13:882147. doi: 10.3389/fphar.2022.882147 (PMC9024123; doi:10.3389/fphar.2022.882147)
Supplement: Supplementary file 2 [file Image1.pdf]

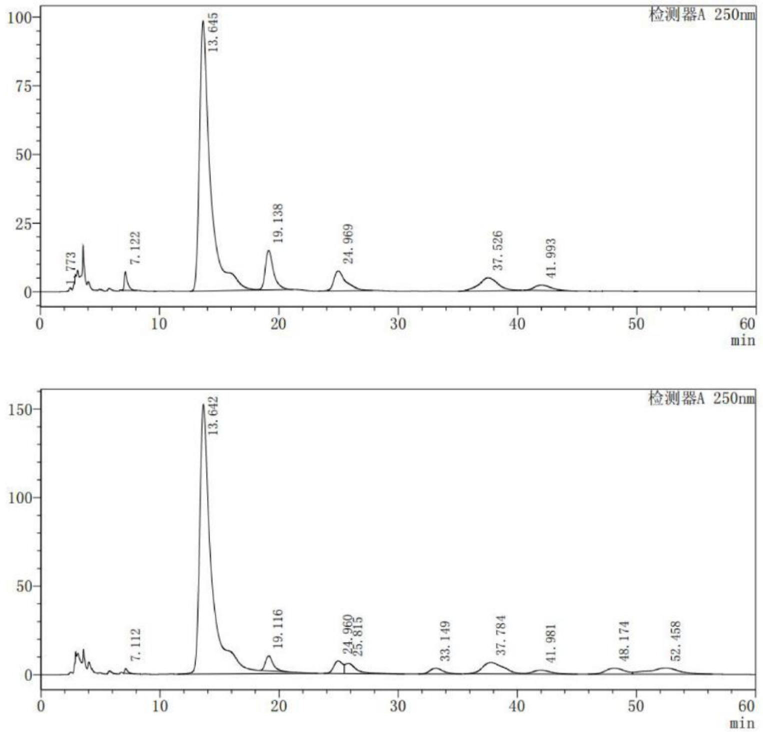

Supplementary Figure 1. HPLC chromatograms of the standard (Top) and HJT (Bottom). The peaks for the standards, from left to right, represent berberine, formononetin, gingerol, ursolic acid, cinnamaldehyde, and anethole.
